# The global landscape of neoadjuvant and adjuvant anti-PD-1/PD-L1 clinical trials

**DOI:** 10.1186/s13045-022-01227-1

**Published:** 2022-02-08

**Authors:** Dawei Wu, Huiyao Huang, Minghui Zhang, Ziwei Li, Shuhang Wang, Yue Yu, Yuan Fang, Ning Jiang, Huilei Miao, Peiwen Ma, Yu Tang, Ning Li

**Affiliations:** 1 Clinical Trials Center, National Cancer Center/National Clinical Research Center for Cancer/Cancer Hospital, Chinese Academy of Medical Sciences and Peking Union Medical College, Beijing, China; 2 Department of Medical Oncology, Harbin Medical University Cancer Hospital, Harbin, China; 3 Department of Basic Medicine and Clinical Pharmacy, China Pharmaceutical University, Nanjing, China

**Keywords:** Neoadjuvant, Adjuvant, Anti-PD-1/PD-L1 treatment, Clinical trial

## Abstract

The neoadjuvant and adjuvant anti-PD-1/PD-L1 treatment has been increasingly noticed. To summarize the global landscape of these clinical trials will provide essential data for all the stakeholders of drug development. Based on the Trialtrove database, a total of 668 clinical trials initiated by the end of 2020 were retrospectively analyzed. We found that a rising capability of global neoadjuvant and adjuvant anti-PD-1/PD-L1 clinical development has been achieved. High prevalent cancer types were extensively studied though the priorities in China and the United States were different. However, a lack of phase III trials and industry-sponsored trials was addressed. The confirmatory neoadjuvant trials were particularly insufficient, and the combination strategy mainly focused on chemotherapy. Thus, more public funding and accelerated regulatory strategies are needed in this field. Efforts should be made to confirm the benefit of neoadjuvant treatment and explore novel combination strategies.

**Supplementary Information:**

The online version contains supplementary material available at 10.1186/s13045-022-01227-1.

## To the editor

Anti-PD-1/PD-L1 treatment is now the standard of care for many cancer types worldwide [1, 2]. Based on the feasibility of checkpoint blockade in the earlier stage of cancer [3–6], neoadjuvant and adjuvant immunotherapy has attracted more attention, especially neoadjuvant settings [3]. However, the evidence on the global panorama of this field is limited. Most of the relevant studies focused on specific cancer types, such as melanoma [7, 8], without the time trend and geographic information. Therefore, we will give a comprehensive analysis of the current pipeline, thus providing essential supportive data for industry, clinical institutions and regulatory authorities.

**Fig. 1:**
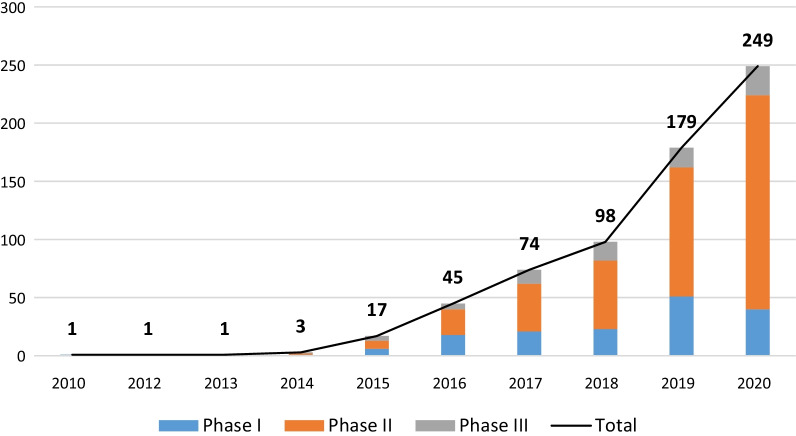
Annual numbers of initiated neoadjuvant and adjuvant anti-PD-1/PD-L1 trials worldwide, overall and by study phase. The compound annual growth rates of overall, phase I, phase II and phase III trials were 73.6%, 44.6%, 91.9% and 71.0%, respectively

Until December 31, 2020, a total of 668 eligible neoadjuvant and adjuvant anti-PD-1/PD-L1 clinical trials were retrieved from the Trialtrove database [9] (Additional file 1: Fig. S1). The annual number of trials showed an upward trend (F = 25.5, *p* = 0.001), with a compound annual growth rate of 73.6%. Phase II trials accounted for the highest proportion (427, 63.9%), followed by phase I (161, 24.1%) and phase III (80, 12.0%) (Fig. 1). There were 433 (64.8%) investigator-initiated trials (IITs) and 235 (35.2%) industry-sponsored trials (ISTs). The IIT was the major type of clinical trials hosted by the United States (149/283, 52.7%) and China (201/216, 93.1%) (Additional file 1: Fig. S2).

Despite the significant increase in trial number, the lack of phase III trials and ISTs suggested that neoadjuvant and adjuvant anti-PD-1/PD-L1 treatment was still at its early exploratory stage. In addition, due to the need for a multidisciplinary team and a prolonged follow-up time, only large pharmaceutical enterprises have the ability to carry out confirmatory registration trials. The country distribution of ISTs is consistent with that of top pharma companies (Additional file 1: Fig. S2). In the future, more experts for multidisciplinary treatment are needed by industries. Policymakers should consider more funding in this field, and formulate accelerated regulatory strategies for the review and approval process, with the application of novel surrogate endpoints, such as pathological response indicators [5, 10].

A total of 24 cancer types were identified in the analysis. Non-small-cell lung cancer (NSCLC), breast cancer (70, 10.5%), esophageal cancer (60, 9.0%) and melanoma (60, 9.0%) were the most common cancers. There were huge differences in the cancer type distribution of clinical trials hosted by the United States and China based on the different clinical needs. Melanoma, breast cancer and urothelial carcinoma that were focused on in the United States, had a relatively high proportion of localized stage at diagnosis [11]. Clinical trials of China mainly targeted esophageal cancer, gastric cancer and hepatocellular carcinoma (HCC), which were highly prevalent and associated with different causes compared to western countries [12] (Table 1).

**Table 1:** Cancer type distribution of neoadjuvant and adjuvant anti-PD-1/PD-L1 trials and the comparison among host country

Cancer type	Host country			
	China	United States	Rest of world	Total
Non-small-cell lung cancer	39	26	35	100
Breast cancer	10	30	30	70
Esophageal cancer	46	8	6	60
Melanoma	10	41	9	60
Urothelial carcinoma	10	29	20	59
Head and neck squamous cell carcinoma	13	29	14	56
Colorectal cancer	16	13	16	45
Gastric cancer	25	11	8	44
Hepatocellular carcinoma	23	7	6	36
Renal cancer	5	10	4	19
Pancreatic cancer	1	14	4	19
Glioma	3	10	2	15
Soft tissue sarcoma	4	6	2	12
Cutaneous squamous cell carcinoma	1	8	2	11
Mesothelioma	0	11	0	11
Ovarian cancer	0	5	5	10
Solid tumor	0	6	1	7
Biliary tract cancer	4	1	1	6
Prostate cancer	0	5	1	6
Cervical cancer	2	2	0	4
Endometrial carcinoma	0	4	0	4
Merkel cell carcinoma	0	3	1	4
Thyroid cancer	2	1	0	3
Small-cell lung cancer	2	0	0	2
Pancreatic cancer and colorectal cancer	0	1	0	1
Gastric cancer and colorectal cancer	0	0	1	1
NSCLC and HCC	0	1	0	1
NSCLC and Gastric cancer	0	0	1	1
Colorectal cancer and pancreatic cancer	0	1	0	1
Total	216	283	169	668

For the treatment mode, on the basis of feasibility including assessing the effect via biopsy of surgical specimen, reducing tumor size before surgery and inducing greater T-cell expansion [3, 6], neoadjuvant mode (544, 81.4%) has attracted more attention. The time trend (Additional file 1: Fig. S3) and geographic distribution (Additional file 1: Fig. S4) of neoadjuvant trials were consistent with that of overall trials. However, among phase III trials, the proportion of neoadjuvant trials (42/80, 52.5%) was not that large (Additional file 1: Table S1). More confirmatory evidence is needed to illustrate the optimal sequence of immunotherapy and surgery, for whether preoperative treatment can bring long-term survival benefits.

Most of the clinical trials were testing combination regimens (554, 82.9%), and chemotherapy was the most commonly used combination partner in both neoadjuvant (286/455, 62.9%) and adjuvant (89/167, 53.3%) phases (Additional file 1: Table S2). Adding PD-1/PD-L1 mAbs to standard neoadjuvant or adjuvant chemotherapy of major cancers was found to be a regular design of combination trials, especially phase III trials (Additional file 1: Table S1). However, in the neoadjuvant phase, combination strategies designed to recruit more immune cells into the tumor, such as immuno-oncology (IO) agents, may be more promising [3–6]. How to expand combination strategies and break through the framework of the traditional neoadjuvant chemotherapy remains to be studied in depth.

In conclusion, the neoadjuvant and adjuvant anti-PD-1/PD-L1 clinical trials have developed rapidly worldwide. High prevalent cancer types with clinical needs have been concerned though the priorities in China and the United States were different. But the clinical development of this field is still at early stage. There are challenges including how to balance the huge cost of clinical operation by public funding and accelerated regulatory strategies, and how to confirm the benefit of neoadjuvant treatment and optimize combination strategies.

## Supplementary Information

**Additional file 1:** Data processing details and additional results.

## Data Availability

All the source data in this work are based on the Trialtrove database, with clinical trial details derived from clinical trial publicity platforms. The datasets used and analyzed during the study are available from the corresponding author on reasonable request.

## Abbreviations

IIT: Investigator-initiated trial
IST: Industry-sponsored trial
NSCLC: Non-small-cell lung cancer
HCC: Hepatocellular carcinoma
IO: Immuno-oncology
