# Supplementary material for: The global landscape of neoadjuvant and adjuvant anti-PD-1/PD-L1 clinical trials
Source: J Hematol Oncol. 2022 Feb 8;15:16. doi: 10.1186/s13045-022-01227-1 (PMC8822713; doi:10.1186/s13045-022-01227-1)
Supplement: Supplementary file 1 — Additional file 1: Data processing details and additional results. [file 13045_2022_1227_MOESM1_ESM.docx]

**Additional file** Supplementary material for

**The global landscape of neoadjuvant and adjuvant anti-PD-1/PD-L1 clinical trials**

Dawei Wu^1†^, Huiyao Huang^1†^, Minghui Zhang^2†^, Ziwei Li^1,3^, Shuhang Wang^1^, Yue Yu^1^, Yuan Fang^1^, Ning Jiang^1^, Huilei Miao^1^, Peiwen Ma^1^, Yu Tang^1^, Ning Li^1^*

*Correspondence: lining@cicams.ac.cn

^†^Dawei Wu, Huiyao Huang and Minghui Zhang have contributed equally to this work.

**Figures**

All the clinical trials registered until Dec 31, 2020

on the Trialtrove databese

Neoadjuvant and adjuvant immuno-oncology

clinical trials retrieved (n=2857)

Neoadjuvant and adjuvant anti-PD-1/PD-L1

clinical trials included (n=668)

Screening with the query (Therapeutic Class is Immunological, anticancer) AND [(Patient Segment contains Adjuvant) OR (Patient Segment contains Neoadjuvant)] AND (Disease contains Oncology)

Observational studies, trials without PD-1/PD-L1 mAbs or surgery excluded (n=2189)

**Number of clinical trials**

- Time trends: overall, by study phase and treatment mode*
- Sponsor type** and host country***
- Cancer type distribution
- Treatment mode and combination strategy

**Identification**

**Screening**

**Included**

**Eligibility**

**Indicators**

**Fig. S1:** Data processing and key indicators

*The treatment mode was defined as the sequence of anti-PD-1/PD-L1 treatment and surgery, namely, neoadjuvant (preoperative treatment with or without postoperative treatment) and adjuvant-only (postoperative treatment) mode.

**The sponsor type was classified as IST or IIT based on the type of the main sponsor.

***The host country was determined by the location of the main sponsor.

**
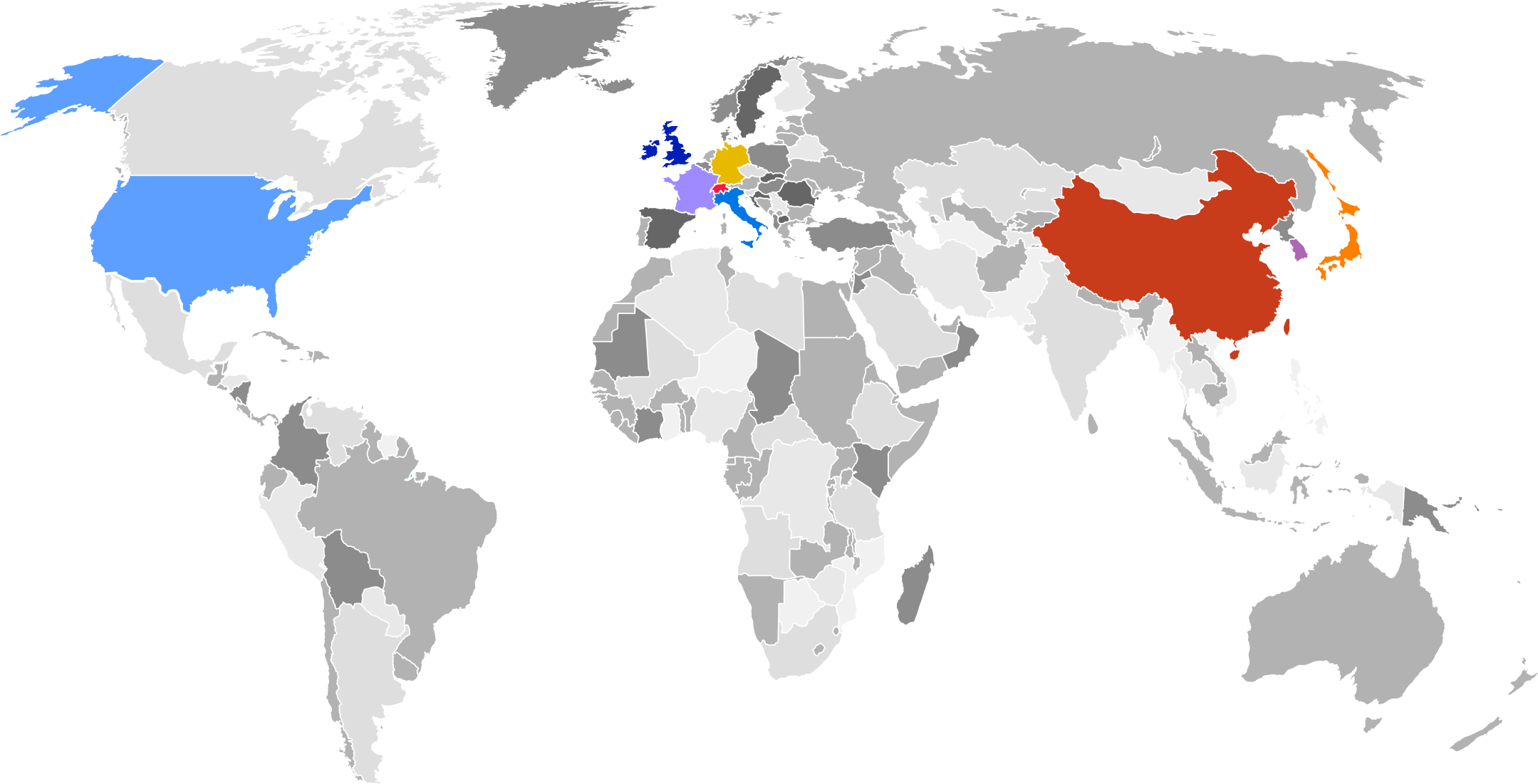
**

**
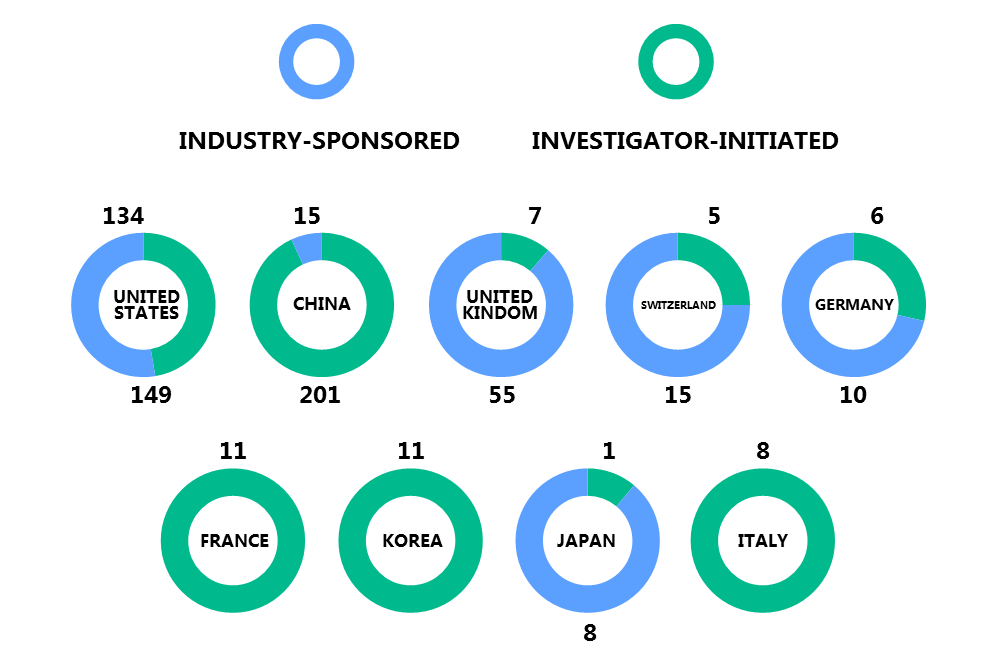
**

**Fig. S2:** Host country distribution of neoadjuvant and adjuvant anti-PD-1/PD-L1 trials by sponsor type

**Fig. S3:** Annual numbers of initiated neoadjuvant and adjuvant anti-PD-1/PD-L1 trials worldwide, overall and by treatment mode

The annual number of neoadjuvant trials showed an upward trend (F=22.7, p=0.001). The compound annual growth rates of neoadjuvant and adjuvant-only trials were 83.7% and 43.5%, respectively.

**
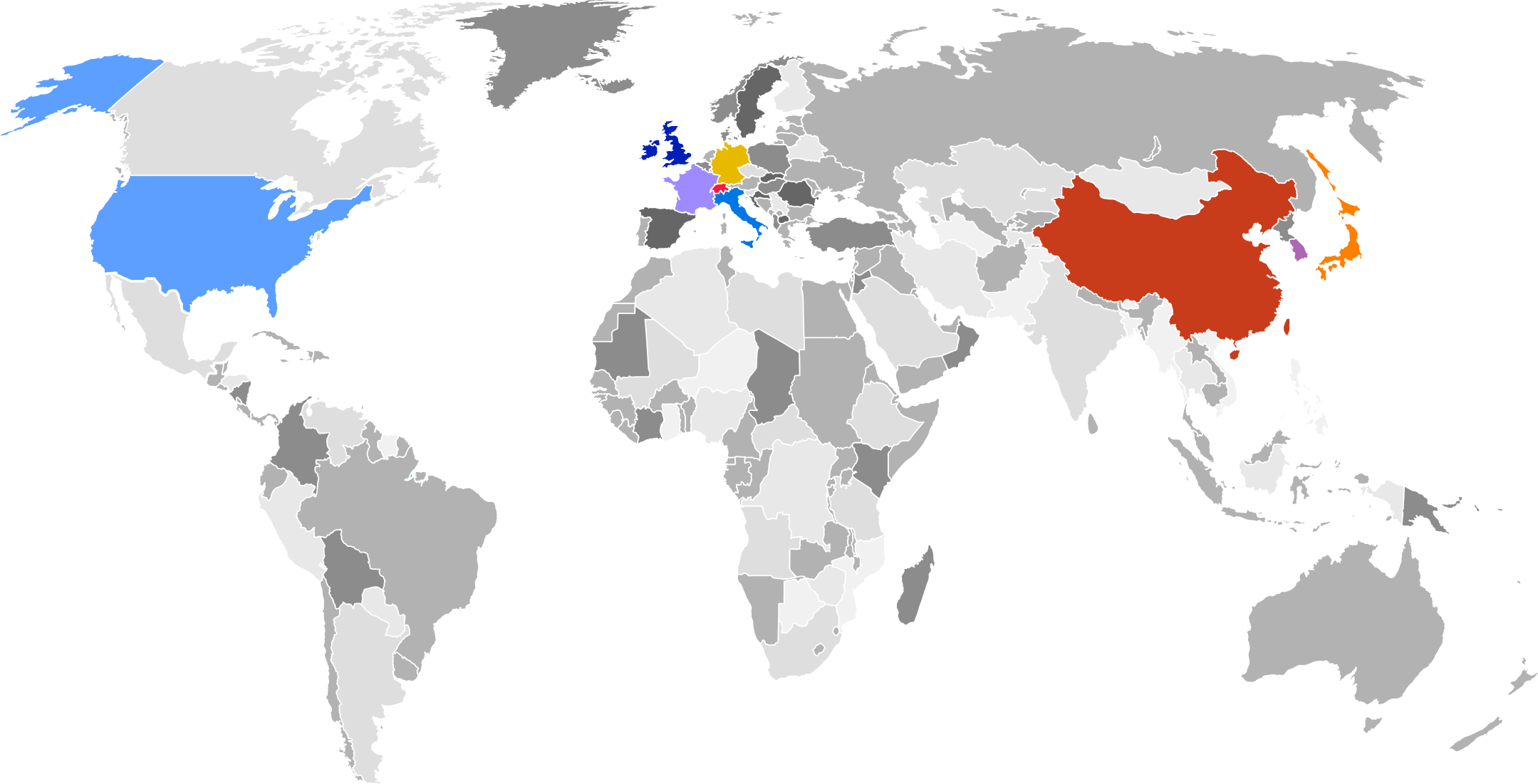
**

**
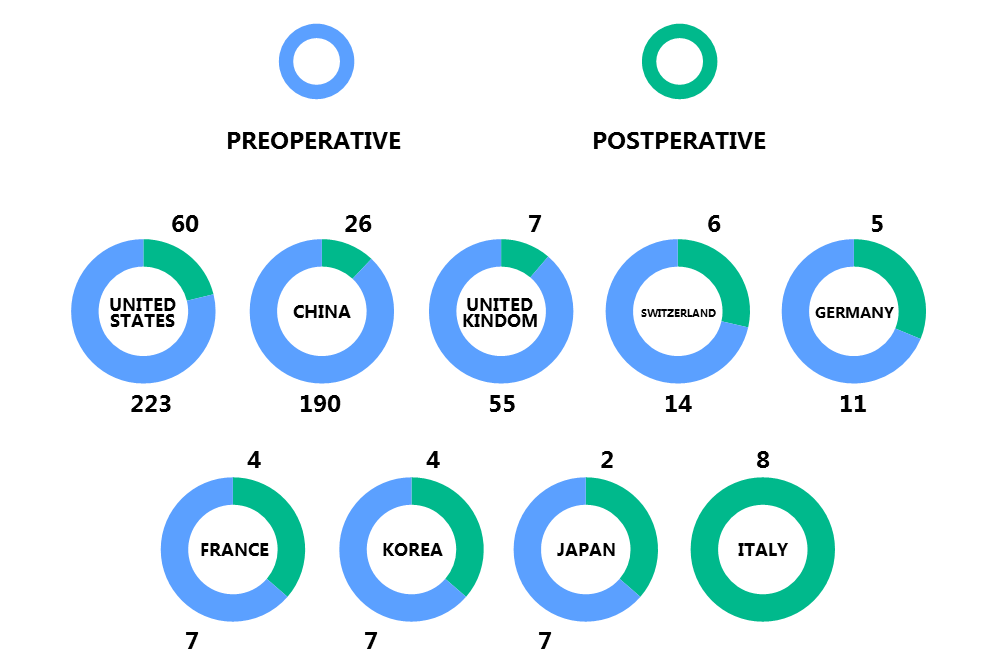
**

**Fig. S4:** Host country distribution of neoadjuvant and adjuvant anti-PD-1/PD-L1 trials by treatment mode

**Tables**

**Table S1:** Cancer type distribution of phase III neoadjuvant and adjuvant anti-PD-1/PD-L1 trials by treatment mode

| Cancer type | Number of phase III trials | | |
| --- | --- | --- | --- |
|  | Neoadjuvant | Adjuvant-only | Total |
| Non-small-cell lung cancer | 11 | 7 | 18 |
| Breast cancer | 12 | 3 | 15 |
| Urothelial carcinoma | 7 | 2 | 9 |
| Hepatocellular carcinoma | 1 | 6 | 7 |
| Melanoma | 0 | 7 | 7 |
| Head and neck squamous cell carcinoma | 3 | 2 | 5 |
| Gastric cancer | 4 | 0 | 4 |
| Renal cancer | 1 | 3 | 4 |
| Esophageal cancer | 2 | 1 | 3 |
| Colorectal cancer | 0 | 2 | 2 |
| Cutaneous squamous cell carcinoma | 0 | 2 | 2 |
| Merkel cell carcinoma | 0 | 2 | 2 |
| Cervical cancer | 0 | 1 | 1 |
| Ovarian cancer | 1 | 0 | 1 |
| **Total** | **42** | **38** | **80** |

**Table S2:** The treatment mode and combination strategy of neoadjuvant and adjuvant anti-PD-1/PD-L1 trials

| Treatment mode/combination strategy | | | No. of trials |
| --- | --- | --- | --- |
|  |  |  |  |
| **Neoadjuvant*** | | | **544** |
|  | **Single-agent** | | **89** |
|  | **Combination** | | **455** |
|  |  | Chemotherapy | 286 |
|  |  | Targeted therapy | 95 |
|  |  | IO | 102 |
|  |  | Radiotherapy | 102 |
|  |  | Other locoregional-therapy | 11 |
| **Adjuvant*** | | | **299** |
|  | **Single-agent** | | **132** |
|  | **Combination** | | **167** |
|  |  | Chemotherapy | 89 |
|  |  | Targeted therapy | 25 |
|  |  | IO | 57 |
|  |  | Radiotherapy | 31 |
|  |  | Other locoregional-therapy | 2 |

*The “neoadjuvant” trials in this table represented the preoperative treatment phase of neoadjuvant trials, while the “adjuvant” trials represented the postoperative phase of both neoadjuvant and adjuvant-only trials.
